# Users’ preferences and design recommendations to promote engagements with mobile apps for diabetes self-management: Multi-national perspectives

**DOI:** 10.1371/journal.pone.0208942

**Published:** 2018-12-10

**Authors:** Mary D. Adu, Usman H. Malabu, Aduli E. O. Malau-Aduli, Bunmi S. Malau-Aduli

**Affiliations:** 1 College of Medicine and Dentistry, James Cook University, Townsville, Australia; 2 College of Public Health, Medical and Veterinary Sciences, James Cook University, Townsville, Australia; Universiteit Twente, NETHERLANDS

## Abstract

**Background:**

Mobile phone applications (apps) offer motivation and support for self-management of diabetes mellitus (DM), but their use is limited by high attrition due to insufficient consideration of end-users perspectives and usability requirements. This study aimed to examine app usage and feature preferences among people with DM, and explore their recommendations for future inclusions to foster engagement with diabetes apps.

**Methods:**

The study was conducted internationally on adults with type 1 or type 2 DM using online questionnaire (quantitative) to investigate usage and preferences for app features that support diabetes self-management and semi structured telephone interview (qualitative) to explore suggestions on fostering engagement and specific educational information for inclusion into diabetes apps. Survey and interview data were analysed using descriptive/ inferential statistics and inductive thematic analysis respectively.

**Results:**

A total of 217 respondents with type 1 DM (38.25%) or type 2 DM (61.8%), from 4 continents (Australia, Europe, Asia and America) participated in the survey. About half of the respondents (48%) use apps, mainly with features for tracking blood glucose (56.6%), blood pressure (51.9%) and food calories (48.1%). Preferred features in future apps include nutrient values of foods (56.7%), blood glucose (54.8%), physical exercise tracker (47%), health data analytics (42.9%) and education on diabetes self-management (40.6%). Irrespective of the type of DM, participants proposed future apps that are user friendly, support healthy eating, provide actionable reminders and consolidate data across peripheral health devices. Participants with type 1 DM recommended customised features with news update on developments in the field of diabetes. Nominated specific educational topics included tips on problem solving, use of insulin pump therapy, signs of diabetes complication and transitioning from paediatric into adult care.

**Conclusions:**

The study has highlighted patients’ perspectives on essential components for inclusion in diabetes apps to promote engagement and foster better health outcomes.

## Background

Mobile phone applications (apps), are extensively used to provide support for diabetes self-management [1, 2]. These apps include features for tracking blood glucose, calories in diet, body weight, as well as reminders for medication intake or health appointments [2–4]. There is strong evidence suggesting that the use of apps encourages adherence to management therapy, improves glycemic control which subsequently prevents or delays the onset of diabetes complications and enhances patients’ quality of life [5–7]. However, despite the proven effectiveness and investments into the technological processes of app development, their use as an intervention for diabetes mellitus (DM) management, has been limited by high attrition rate [8–10], evidenced by reduced user engagement over time. The low level of adoption and use of apps has been attributed to insufficient consideration of end users’ preferences [2, 11] and the factors for engagement [12].

### Trends in users’ preferences for features and engagement with apps to support diabetes management

There are limited studies that have explored patients’ use, feature preferences and recommendations that could improve engagement with diabetes apps. Recent surveys found that although many people with DM own a smartphone, only a few of them used apps to manage their diabetes [13, 14]. The major reason for non-use was that patients were unaware of the existence of apps that could support their care [14]. Additionally, there are diverse patients’ views about the essential features in apps to support diabetes management. A survey found that features for data recording, social coaching, reminder and remote collaboration with health care professionals were appealing to people with DM [13]. Other studies have reported carbohydrate counter, blood glucose and physical activity tracking as the most commonly preferred app features by diabetes patients [15, 16]. However, most apps were unable to meet up with patients’ needs because diabetes education which is a crucial and evidence-based requirement for diabetes management is often lacking in the currently available apps [2, 17].

Moreover, to be an effective self-management support tool, app must continuously capture the attention of users and stimulate users’ interest to actively engage with it. Engagement indicates the degree of interaction a user has with the technology within a given time span or the overall length of time from the onset use of a technology to when the user totally lost interest in the usage [18]. Engagement with health technologies including apps is a dynamic process comprising of different stages, namely: point of engagement, period of engagement, disengagement and reengagement. Therefore, users’ engagement is multifaceted in nature and may change within a short or long periods of time [19]. User’s engagement can either be measured during a short session or long-term use of a technology. It is important to note that the ultimate goal of mobile apps usage for patients with chronic diseases is to foster their ongoing and regular participation in their self-care activities. Where participation in those activities may be reflected in the frequency of tracking or monitoring of the activities using an app hence denoting the extent of their engagement with the app. Nevertheless, patients may participate in self-care activities and not track with an app (non-usage) [13] even when apps are present on their mobile devices, which may be due to several reasons. Since health technologies are voluntary use systems, the extent of engagement with them is determined by the users’ perceived quality of experience, ongoing benefit of usage [20], consideration of viable alternatives to using the technology and decrease in perceived costs [18]. Furthermore, Studies have reported that mobile health apps which are able to adequately meet patients’ self-management needs and have clear evidence of data privacy will motivate users engagement with the technology [21, 22].

Low engagement is not unique to diabetes apps alone, but rather to all computerised behavioural therapeutic interventions that support the management of chronic diseases or health promotion [23–26]. Engagement with mobile apps is negatively affected by factors such as lack of motivation or commitment to change health behaviours [23], and knowledge in managing the targeted behaviour. Once knowledge is attained, it is likely that participants’ interest in app use will reduce [24, 27]. Moreover, sub-optimal usability has been stated as a reason for low engagement with apps [28, 29]. Optimal usability in apps could be achieved through simplicity, reduced time consumption [5, 30] and customized users’ experience [31]. To date, there is little or no clear evidence as to why engagement with diabetes apps remains low. Tatara et al 2013, in their study on long-term engagement with a mobile self-management system for people with type 2 diabetes reported perceived sense of mastery over diabetes and experiences of problems with the app as factors for declining motivation to continuous use of app [32].

The few studies described above have shown that diabetes patients have different needs and requests for health care technology development [33] and there is limited understanding of the factors which could foster engagement with apps to support diabetes. Moreover, these studies have been limited to single countries and current app users alone. A critical step to knowing what appeals to end users and maximizing app engagement is shared decision-making, whereby app developers involve targeted end users in the process of developing the content and features of apps [12, 34]. Hence, it is important to build upon previous research by examining the perception of a diverse range of people with diabetes; that is both current users and non-users, residing in diverse locations, about the usability and functionality of diabetes apps to support their health-care and factors for usage over time.

### Study aims

Thus, the aim of this study was to involve individuals with type 1 and type 2 DM in guiding the development of future mobile apps for diabetes self-management. The specific objectives were: (1) to assess the use and preferences for potential features in apps which could support the health management needs of individuals with type 1 or type DM. (2) Seek recommendations on components and motivators that could foster long term use of apps (3) Assess specific educational topics desired in apps to support diabetes management. We hypothesized that diverse multinational respondents comprising of adults with type 1 or 2 DM (both users and non-users of apps) would recommend the necessary design components and strategies to maximize engagement with apps to support the self-management of diabetes. Getting this insight would provide an effective approach to the design and development of an evidence-based and highly functional app that best meets the identified needs of people with diabetes.

## Methodology

### Study design

The study employed a mixed methods design, combining quantitative and qualitative data to explore patients’ perceptions and the results were integrated in the data interpretation phase [35]. Information was sought globally from adults aged > 18 years who have type 1 or type 2 DM. Users and non-users of apps were included in the study to obtain balanced and un-biased findings.

#### Quantitative approach

Data were collected through an online survey conducted between November 2017 and June 2018. The study was advertised using multiple outlets, including Townsville Bulletin digital newspaper, Diabetes Australia and Diabetes UK websites as well as various diabetes support groups on Facebook and Twitter. Through these advertisements, a link was provided where potential participants were directed to the Survey Monkey page containing information about the study. Consenting participants could subsequently click on the survey link and submit their responses. As an incentive to encourage participation, a chance to enter a draw to win one of 6 US$50 e-gifts card was offered to respondents. This recruitment procedure yielded an inadequate response rate particularly from Asia and Europe, hence the need for another approach. More targeted respondents were recruited from Asia and Europe, and were offered an additional incentive ($US 5) for their participation.

#### Instrument

Through a systematic review, an overview of the frequent features currently available in mobile apps to support diabetes management as well as the gaps in literature were obtained [36]. This review guided the development of the study questionnaire which comprised two sections. The first section inquired about basic demographic and health characteristics of the respondents. The second section comprised 3 questions aimed at elucidating preferences and perceived importance of various features in apps that could be utilized by patients with type 1 and type 2 DM, regardless of the use of insulin as part of the management therapy. The first question which was on a dichotomous scale (Yes or No) inquired about the current use of apps to manage DM. Those who indicated ‘No’ were requested to specify reasons for non-use while those who answered in the affirmative were asked to specify the features available in such apps. The second question assessed participants’ preferences on a list of app features and allowed multiple responses. The third question evaluated the perceived usefulness of various app features to support diabetes management. These features were rated on a 10 point scale with ‘‘1 = least useful and 10 = most useful”, so that higher rating indicates greater perceived usefulness. Descriptors for intermediate points within the range were excluded to avoid clustering of scores around a preferred descriptor. App was defined as any program downloadable to a smart phone which is used to support any aspect of diabetes management to foster improved health outcomes for the user. It could either be lifestyle oriented such as those for tracking diet and exercise or patient oriented for blood glucose or blood pressure monitoring. The instrument was reviewed among a diverse team of researchers with expertise in survey development in order to ascertain its readability and ease of understanding, and was revised based on feedback obtained. The instrument readability was further accessed with the Flesch Kincaid readability test [37] producing a reading score of 61.8, indicating the instrument is well comprehensible, consistent with standard English that is easily understood by an eighth or ninth grade level student or one who has attained 13 years of age or above [37]. The instrument was administered in English Language. The final instrument used in the survey is available in S1 Appendix.

#### Qualitative approach

Semi-structured telephone interviews were conducted with a subset of participants who responded to the questionnaire. To recruit participants for the interviews, a final question was added to the questionnaire asking for interest in a telephone interview aimed at further exploration of opinions on the use of apps to support diabetes management. Interest was indicated by providing a phone number and suitable contact time. No additional incentive was offered to interview participants. A standardized text message was sent to all phone numbers provided to confirm interview schedules. All interviews were conducted in June, 2018 by a resource person who was independent of the research team (designated as the interviewer, who is a male researcher with prior experiences in qualitative research and interview facilitator). The interviewer was trained on the aims of the study and the interview guide by the first researcher (MDA). Additionally, a pilot session of the interview was conducted between the interviewer and MDA. The interviewer had no prior relationship with the participants and the interview sessions was conducted in a secure, private room at the James Cook University, Australia. Prior to the commencement of each interview session, participants were asked if they were in a location that was convenient for the interview. Interviews were conducted in English and MDA was present during the first three interviews to listen to the interactions between the participants and the interviewer and to ensure appropriate data acquisition. There was no previous relationship or interaction between MDA and the participants. Data saturation was achieved at the end of the 12^th^ interview, that is, the information was deemed sufficient as there were no new response patterns identified by the interviewer at this point. However, interview sessions with all consenting respondents (16) were completed in order to allow rich documentation and to ensure no point was accidentally missed. Repeat interviews were not required. Given that participants’ location to the research site was remote, we were unable to return data transcripts to participants for comments.

The interview questionnaire was developed and iteratively reviewed by the research team. The notion behind the development of the questions was to seek recommendations on components and factors which patients considered important to continually stimulate their interest in the regular use of apps. Based on the fact that health technologies are voluntary use systems, therefore, the extent of engagement with them is determined by users’ perceived quality of experience, ongoing benefit of usage [20], and consideration of viable alternatives to using the technology [18]. Furthermore, the development of questions on specific educational topics which could be incorporated into diabetes apps was informed by these reasons: (i) gaps in the literature which shows that most of the currently available diabetes apps are lacking in educational component [2, 17, 36] and (ii) the ability of apps to meet self-management needs (diabetes education fosters self-care); which could subsequently motivate users’ engagement with apps [21]. Refer to S2 Appendix for interview guide.

### Ethical considerations and consent

Approval for the study was granted by the Human Research Ethics Committee of the James Cook University (H7087). Submission of the survey responses implied consent to participate in the quantitative phase of the study while verbal consents were obtained for the telephone interviews and recordings.

### Data analyses

Quantitative data analysis was done using SPSS version 23 (IBM, Armonk, New York, US). Continuous variables were reported as means and standard deviations (SD) while categorical variables were reported as percentages. Chi squared test for independence and ANOVA were used for group comparisons involving categorical and continuous variables respectively. Statistical significance was set at *p* < 0.05

For qualitative data analysis, each interview was digitally recorded and the duration ranged from 7 to 20 minutes. Audio files were professionally transcribed by an individual with privacy certification and reviewed for completeness. Raw data files were imported to QSR Nvivo 11 for open coding and analysis. Emerging themes were identified using an inductive thematic analysis approach in order to ensure rich description of the data set [38]. The thematic data coding followed six phases as described by Braun and Clarke (2006) [38]. (1) Familiarising with the data through reading and re-reading the transcripts to make meaning of participants’ responses; (2) generation of initial codes; (3) initial coding to identify emerging themes; (4) review of identified themes; (5) refining theme names and (6) documenting the findings and presenting illustrative quotes from participants’ responses to support each theme. All phases of the thematic coding was completed by a researcher (MDA). To establish the trustworthiness and credibility of the data, second researcher (BMA) was involved in phases 2–6 of the thematic analysis. Data were cross-checked in a consensus meeting; there was an 80% agreement between both researchers and discrepancies were resolved through discussion and mutual agreement. Both MDA and BMA have prior methodological training and experiences in conducting qualitative research. The remaining two researchers also checked the codes and themes to ensure consistency. Illustrative quotes were appended with a combination of number code and the type of diabetes the respondent has (for example ‘‘P4, T1D”). Quotes which included marketed names of apps were not reported verbatim for commercial reasons. The final manuscript was reported according to the COREQ criteria for reporting qualitative research [39] (See S1 Checklist).

## Results

### Participant characteristics

A total of 245 respondents attempted the survey. Twenty-eight responses were excluded due to missing data (19) or ineligibility where the type of diabetes was not stated (9), leaving a total of 217 complete responses for the analyses. Participants’ mean age was 44.65 (SD 11.51) and ranged between 18–76 years. They were predominantly type 2 DM (61.8%), females (55.7%) and were diagnosed with DM within the previous 1–5 years; (40.1%). Further demographic and health details are provided in Table 1.

**Table 1:** Demographic and health characteristics of survey respondents.

	Study Participants (N = 217)	
Characteristics	n	%
Gender		
Male	94	43.3
Female	123	56.7
**Age (Years)**		
18–29	27	12.4
30–39	64	29.5
40–49	41	18.9
50–59	46	21.2
60–79	39	18
**Education**		
High School	44	20.3
Technical College	41	18.9
Bachelor Degree	60	27.6
Post Graduate degree	62	28.6
Others	10	4.6
**Continent**		
Australia	75	34.6
Europe	76	35
Asia	64	29.5
America	2	0.9
**Employment Status**		
Employed	148	68.2
Unemployed	36	16.6
Retired	33	15.2
**Type of Diabetes**		
Type 1	83	38.2
Type 2	134	61.8
**Duration of Diagnosis (Years)**		
< 1	27	12.4
1–5	87	40.1
6–10	44	20.3
11–15	16	7.4
> 15	43	19.8

For the telephone interviews, initially 31 respondents indicated interest to participate by providing phone numbers on completion of the survey. However, due to subsequent decline, only 16 respondents participated in the phone interviews. Respondents were predominantly males; 56.2% (9/16), Australian residents; 87.5% (14/16) and have type 1 DM; 62.5% (10/16). Their ages ranged from 26 to 61 with a mean of 44.56 (SD 11.51) years old.

### Use and non-use of apps

Less than half of the participants (106/217, 48.8%) used apps to support their health management, where blood glucose tracker (60/106, 56.6%), blood pressure tracker (55/106, 51.9%) and food calorie counter (51/106, 48.1%) were the mostly used features. The remaining 51.2% (111/217) of the respondents did not use apps to care for their DM. Major reasons for non-use were ‘‘lack of awareness” (52/111, 46.8%) and ‘‘disinterest” (52/111, 33.3%) in the use of apps (See Table 2 for details).

**Table 2:** App use, preferences and perceived usefulness of app features to support diabetes management.

Variables	n	%
**Usage of App [N = 217]**	** **	
Yes	106	48.8
No	111	51.2
**Features in apps [n = 106]**		
Blood pressure tracker	55	51.9
Blood glucose tracker	60	56.6
Food calorie counter	51	48.1
Fitness/exercise monitor	50	47.2
Body weight monitor	35	33
Transfer of health data to doctor	20	18.9
Reminder (e.g take medication, BG^a^ monitoring)	38	38.6
Others^b^	10	9.4
**Reasons for not using app [n = 111]**		
Not interested	37	33.3
Lack of awareness	52	46.8
Do not have a smart phone	12	10.8
Lack of access to the internet	8	7.2
Expensive	10	9
Missing	10	9
**Preferred features / functions if offered a new app [n = 217]**		
Food nutrient composition	123	56.7
Blood glucose tracker	119	54.8
Body weight tracker	79	36.4
Physical exercise tracker	70	32.3
Task reminder	70	32.3
General education on DM self-management & complication prevention	88	40.6
Personalized advice /education in response to logged BG data	87	40.1
Visual Analytics (view trends of logged data)	93	42.9
Data export to Doctor or other health team members	75	34.6
Social networking among people with diabetes	54	24.9
**Apps feature ratings**	**Mean**	**SD**
Blood pressure tracker	6.64	2.7
Blood glucose tracker	8.35	2.03
Fitness and exercise monitor	7.37	2.29
Body weight monitor	6.93	2.45
Task reminder	6.71	2.56
Export of data to health care team (e.g doctor)	6.91	2.7
Social networking	5.96	2.99
Food nutrient composition	7.73	2.32

^a^Blood Glucose

^b^bolus insulin dose calculator, heart rate monitor, apps for continuous glucose monitors

#### Influence of participant characteristics on the use/non-use of app

App use was significantly influenced by demographic/health characteristics. Individuals more likely to use apps tended to be younger (*p* = 0.00), have type 1 DM (*p* = .002), resident in Asia (*p* = .000), employed (*p* = .001) and have higher educational level (*p* = .0.00) than their counterparts in their respective demographic domains.

### Preferences for app features and perceived usefulness

When asked to indicate the features they would prefer in a new app for diabetes management, major participant inclinations were towards apps with information on nutrient value of foods (56.7%), blood glucose tracker (54.8%), physical activity tracker (47%) and visual analytics to view trends in health status indicators (42.9%). Furthermore, interests were expressed in apps that provide either general education on diabetes self-management (40.6%) or personalized education in response to logged blood glucose data (40.1%).

Participants’ ratings of perceived usefulness of app features to support diabetic’ health management were highest for blood glucose tracker (mean 8.35 [S.D 2.03]), food nutrient counter (mean 7.73 [SD 2.32]) and fitness/exercise monitor (mean 7.37 [S.D 2.29]). Apps for social networking among people with diabetes (mean 5.96 [S.D 2.99]) had the lowest rating. (See Table 2 for details).

#### Influence of participant characteristics on preferences for app features

App feature preferences were not influenced by type of DM. Affirmative response rate for each of the suggested app features varied between 25-48/83 (30.1–57.8%) for those with type 1 DM and 28-76/134 (20.9–56.7%) for those with type 2 DM. The most commonly preferred feature by all participants was food nutrient composition (56.6%, 47/83 for T1DM and 56.7%, 76/134 for T2DM). Also, 54-123/217 (24.8–56.7%) of all respondents had an inclination towards each of the various suggested features, irrespective of DM type.

### Recommendations to foster long-term engagement with diabetes apps

Four themes emerged from the interview based on recommendations to foster long-term engagement with diabetes apps. The themes include: (1) improved functionalities (exciting and new recipes, actionable goals with convenient reminder), (2) certified and reliable information sources (social networking, research and news update), (3) consolidated and customized features, (4) ease of use. The themes and subthemes are reported below with representative quotes.

#### Theme 1: Improved functionalities

**Subtheme1.1: Exciting and new healthy recipes**:

Participants commonly advised that essential attributes which could foster their engagement with apps that support healthy eating was for such apps not to offer just suggestions on suitable food recipes for people who have diabetes, but that the recommended recipes are also delectable:

"Some ideas about recipes to brighten up my day, because turning food into just fuel I think is just terrible. Although, we do have to kind of trim off some lavish recipes, but that doesn’t mean it has to be eating cardboard". [P3, T2D]

Some participants want healthy eating support by apps to include assistance with shopping:

"I was thinking like- just look up something. If you are in the supermarket and you decide what you’re going to buy, if I haven’t prepared my menu, it would be nice not just to rely on memory all the time and be able to look up something (in the app), like something different to make". [P8, T2D]

Additionally, many participants felt that when apps provide nutritional information not only on various conventional foods but also on rare foods, this could stimulate their continual use:

"The aspect with just kind of having all available, so more not so common foods and you can virtually put any product in (the app) and it will split out the nutritional table". [P9, T1D]

**Subtheme 1.2: Actionable goals with convenient reminders**:

Most of the participants said, they would be highly attracted to engage with the use of apps for their self-management if its’ reminder feature can be complemented with actionable instructions on how to accomplish behavioural goals of diabetes self-management:

"My anxiety and stress levels are high around the time of my menstrual period, so I find my sugar levels difficult to manage around that time…So if an app could connect to my CGM and sort of warn me about those days. So if those could be tracked and I could be given a reminder thing like, okay, you need to cool down, you need to go for an extra session of meditation, or you need to run a little bit more during this time, that would be helpful". [P11, T1D].

A participant who identifies as a software developer gave specific examples of how apps could provide actionable reminder:

"Actionable knowledge that nudges me on the key items which helps me to succeed. For example, I’ve been messing around with the idea of building an app that geofences the bad places that you shouldn’t eat. I mean first of all, geofence every single [Name of an American fast food company], so right from the moment you walk into them, it pings up and say you don’t really want to be here…ordering this food, this is a bad thing, at this time" [P3, T2D].

"It can be listen, I’ve looked at the number of steps that you walked and you need to do… go for a walk at lunch time otherwise, you’re not going to do and you know you that you’ve got to do something active for at least 30 minutes a day to help your diabetes". [P3, T2D].

Nevertheless, some participants commented that in the event that a user is unable to immediately review a behavioural data prompted from an app, it is necessary that users are able to turn off such prompting to attend to it at a more convenient time:

"Because you don’t always want to be nagged by these things. Sometimes that sort of prompting-I mean there are apps that will automatically remind you, if you had a low blood glucose they might go, they might check back in and they might remind you in half an hour, or you might be able to set an optional reminder, to check again and make sure it is resolved. But you don’t want any of that functionality to be so hard wired that you can’t turn it off, because sometimes it’s not convenient just at the moment. You know you might be in a meeting or you just never want to be nagged like that". [P14, T1D]

#### Theme 2: Certified and reliable information sources

**Subtheme 2.1: social networking**

In multiple instances, participants, particularly those who have type 1 DM, emphasised that interfacing of apps with social forums would be appealing. They would like to make social connections using apps, through which they can access role model narratives of stories from other individuals who had overcome salient obstacles in diabetes management. Participants believed that social networking in apps could serve as a motivator for long-term engagement as well as an opportunity to receive information on problem-solving:

"I think personal experience would be a massive thing. If I could sit down and read someone else’s story and how they’ve gone from what I’m at now, like with minimal control, to being on top of it. It would be awesome to hear from someone that’s done it, to give me a belief that I can do it. An app could be the only way to sort of get that connection ". [P12, T1D]

Participants further explained that apps linked to certified information sources would equally serve the similar purpose of providing a platform for accessing problem solving techniques in diabetes management:

"For apps to be able to make connections to other information sources will be good. So if for example, that you-it was a logging app and it could be seen that the frequency of low blood glucose had increased this month…., may be you may be directed out to resources that might help someone think about why that is. You know, either, peer-to-peer resources or respected kind of academic resources on ways to manage whatever the challenge is". [P14, T1D]

**Subtheme 2.2: Research and news update**

Many participants with type 1 DM requested that apps should serve as a resource for news update on ongoing developments in diabetes management. The participants want an app to function as a platform to seek out information whether in form of recent research, technology, medication or treatments which could further improve diabetes care:

"Good information on the latest technology or when the next pump is going to be approved. Am constantly looking for something that is going to make it (diabetes management) easier. It can go in there (into an app) and it’s going to tell me what’s waiting for FDA approval or what’s the latest thing". [P1, T1D]

"In particular, the latest research and things they are implementing in recent times. So the latest news on use of medications and even new treatments would be fabulous (in an app)" [P7, T1D].

#### Theme 3: Consolidated and customized features

**Subtheme 3.1: Data consolidation**

Consolidation of data from peripheral health devices used by patients into a single app was also an important consideration. Participants frequently mentioned that getting merged data would provide a unified record view:

"So it’s got to be integrated completely into the health data in the phone. It can’t be a stand-alone. It’s got to be sharing data with everything else, so that if I look at my consolidated health chart collected by the app, say, how much I drank and may be the total sugar count I’ve consumed, I kind of want to see the (combined) data collected" [P3, T2D]

Also, participants’ felt that when data are consolidated, clear meaningful outputs will be provided which will remove the barrier of deciphering the relationship between the data provided on each of the various health devices:

"Well, it’s actually linking all the information together. I’m on a Continuous Glucose Monitor. I’m on a pump and I have the Fitbit. Trying to link all these three things together is impossible. What I would like is for all the information to correlate with each other and be able to see it potentially on a single page" [P4, T1D]

Some participants noted that non-interdependency of data from various health devices is difficult to manage especially for people with low education and may discourage their use of app:

"You know, you’ve got products like (‘‘name of app”) and (‘‘name of app”) for booking doctors’ appointments. So I’ve got the (‘‘name of app”) managing my CGM, I’ve got insulin pump managing my insulin injections and telling me how much carbs I’ve had to how much I need. Then, I’ve got different system to manage doctors and different systems to manage my carb counting. So, it’s complex and if you’re not literate enough to be able to use your phone in that way, then it’s a big negative. So you could have that integrated solution". [P2, T1D]

**Subtheme 3.2: Customized features**

Participants with type 1 DM stated that the ability of apps to adapt to changes in users’ requirement could encourage adoption and enhance engagement. Participants indicated that they would like to engage directly with specific functionality they desire to use per time without interruption by other functionalities which may be present in the same app:

"I think the thing to bear in mind is management and management strategies vary significantly from person-to-person. If you have an app that’s got 15 functions, each individual person might really only use six or seven of these. For example, I’ve got no interest in calorie at all and some other things and someone might be really interested in keeping track of their blood pressure. Again it’s not something am particularly interested in. So the fact that it (the app) supports that is great but for me I’d want to be able to turn that entry thing off on the screen so I haven’t got to keep scrolling past stuff am never going to be using. So, it’s the tailorability, it’s making it flexible, really". [P14, T1D]

Additionally, inability of apps to adapt to the needs of a user could serve as a barrier to usage:

"Less amount of clicking stuff you’ve got to do on the app, I suppose would be beneficial. I like getting my tester out and all that stuff. Then having to go into an app where I’d have to go through 30 different things to find what I’m looking for would be more of a hindrance than what it would be to not just use it at all" [P12, T1D]

#### Theme 4: Ease of use

Several of the participants pointed out that simplicity of usage and limited time consumption by apps are major determinants for their engagement with the app. For example, a participant explained his view on these facts by giving reasons for abandoning a specific app for the other:

"I was formerly using (‘‘name of app”). I just couldn’t get it so I stopped using it. So the one I use now (‘‘name of app”) as a carb counter is just a bit easier to use and not too time consuming" [P1, T1D].

Apps ability to save and display previously logged data will also limit the time requirement for use:

"Apps that remembers what you have put it,…because you will probably find that we eat a lot of same foods all the time but having to re-enter that in as a new-entry every single time is a bit of pain". [P2, T1D].

### Specific educational topics desired in future apps to support diabetes management

Three major topics emerged from participants’ responses to the question on areas of diabetes management which may be embedded as educational information into apps. The topics were approaches to problem solving, basic guidelines for the management of DM (transitioning from paediatric to adult care, signs of acute and chronic complications of diabetes) and the use of insulin pump therapy.

#### 1: Approaches to problem solving

Majority of the participants proposed that apps should provide information on specific steps to take in response to challenges posed in the self-management of diabetes. Specifically, respondents expressed their desire for apps to offer education on possible strategies for problem resolution when logged blood sugar data are out of the clinically recommended range:

"I think you need to potentially—if someone is put in low blood sugar or a high blood sugar, you need to give directions on what they need to do…whether you need to have more insulin or if you are low, you need to have 15g of carb.. just pointers like that would potentially help people’s management longer term" [P4, T1D].

Furthermore, automating the problem solving suggestions, with the inclusion of information on the health consequences of blood glucose levels per time was recommended by respondents as echoed in the quotes below:

Just putting in whatever the blood sugar reading might be, and it could come back with ways to help me with that". [P8, T2D]

"Let’s say I did a pinprick (blood sugar) test and I had a reading that was odd, I would like to see what the implications of that is". [P8, T2D]

#### 2: Basic guidelines for the management of diabetes mellitus

2.1: Transitioning from paediatric to adult care

Participants with type 1 DM indicated that the inclusion of information to augment their knowledge of self-management during the transitioning phase from paediatric into adult diabetes care would be helpful. Participants find this phase of life to be difficult, thus, they mentioned that providing encouragement/teaching for self-care skills via an app may help to answer some of the mind-boggling questions they have about their management:

"I think mainly, issues when you become a bit older. So, it’s just that middle section, I found it quite difficult going through transition, so from probably about 18 until about 30. So information as you leave adolescence into adulthood. Also, going into pregnancy, there are a lot of questions I’ve kind of like asked out of curiosity to my endocrinologist about pregnancy, which I was quite shocked at the answer". [P9, T1D]

2.2: Signs of acute and chronic complications of diabetes

Many participants noted that apps having information on the signs of acute and chronic complications of diabetes would further create awareness about the disease and the importance of self-care:

"I think it would definitely be beneficial to have (information) on the problems and complications. Like what problems you will face before you face the major complications, so you sort of know what to look out for and what to be aware about". [P12, T1D]

The importance of the reliability of all provided information in apps was also emphasised:

"From a lot of people that I’ve spoken to, everyone get different information from different doctors…doctors have got medical view which they must pass across and they’ve got their own personal view. So I would say, it’s got to come from the Australian standard, you know medical view of diabetes ". [P6, T1D]

#### 3: Use of insulin pump therapy

Provision of guidance on insulin pump therapy through apps was highlighted by many participants with type 1 DM. The participants enunciated that having adequate knowledge on how to set a pump to deliver the right amount of insulin (especially bolus rate) could be challenging. Therefore, they expressed that having information on the use of insulin pump therapy in apps would be beneficial to foster the attainment of a stable blood glucose levels in them:

"Something like (when to deliver boluses on insulin pump). I mean, it’s potentially something that is quite hard to do…even when you have a pump before a meal is actually not really good thing to do because your insulin starts working at the same time you are eating. If you dose 15 minutes or 30 minutes before you eat, you then don’t get a peak because you’ve got your insulin working at the same time as the food is being digested. That sort of stuff- long term for a diabetic I think is critical so you are not going up…you are not doing the rollercoaster". [P4, T1D]

It was further stated that guidance on physical exercise and insulin dose adjustment when using a pump therapy would be helpful. Participants want apps to provide direction on administration of basal insulin relative to glycemic levels in order to reduce or prevent hypoglycaemic events:

"So my experience about 6 months ago was that I started exercising and after 15 minutes of crunches I hit a 3 from 10 sugar level. I had my pump disconnected at that point. So I’ve been a little scared to get back to that. So in this scenario, it would be helpful if I sort of then put my sugar level in (an app), when I’ve exercised last or how am feeling at this point and I’d get at least an idea of whether I should continue to use the pump or disconnect it altogether". [P11, T1D]

## Discussion

A mixed methods study (online survey and telephone interviews) was conducted with a diverse pool of participants who have type 1 or type 2 DM. The online survey focused on obtaining information about the use and preferences for app features which could support diabetes management. The interviews provided recommendations on how to foster long-term engagement with apps that support diabetes management and the educational topics which could be included in such apps.

### App use and preferences

Statistics on current app uses (48.8%) and mostly used app features (food calorie counter, blood glucose and blood pressure tracker) are in congruence with previous studies [15, 25]. Monitoring of blood glucose and blood pressure are important to attain diabetes management goals, therefore inputting these data into apps may encourage personal reflection and reveal out-of-range values in need of urgent treatment.

The current study also echoes the findings of previous surveys which reported that usage of apps were mostly seen among individuals with type 1 DM, those of younger age, high educational level [16], employed and resident in Asia [26]. These observations confirm that socio-demographic characteristics of individuals especially the age, level of education and income status have a strong influence on their digital health use [40–42]. Additionally, eHealth literacy (the potential to accurately interpret health data obtained from mobile health devices) has a direct correlation with the aforementioned socio-demographic characteristics [43], thus, may also be responsible for the app usage trends observed among the sub-groups in this study.

Contrary to the findings of other studies on app use among people with DM [13, 44], a larger proportion of the respondents in this study do not use apps, primarily due to lack of interest and awareness. Given that most patients with chronic diseases often regard regular tracking of their health as an additional burden [45], it is unsurprising that lack of interest was a major reason for not using an app. It is therefore important that apps are unambiguous, provide clear information on the specific health benefits for patients and at the same time are intuitive enough to stimulate interest in usage. Furthermore, since the use of apps offer great potential to support diabetes management, clinicians may promote and create awareness about apps to patients through their unique role of providing health recommendations to their patients [46].

Interestingly, the type of diabetes participants had did not influence their choice of app features. Preferences and ratings were highest for information on nutrient content of foods and capacity to track blood glucose. This result is expected since healthy eating and regular monitoring of blood glucose are important self-management domains for people with type 1 and type 2 DM in order to ensure healthy life [47]. Due to the direct impact of food on blood glucose levels, monitoring carbohydrate and calorie intake is imperative to maintain optimal glycemic control [48]. Therefore, people with diabetes may benefit from apps that help with nutritional tracking in order to assess the impact of foods on blood glucose levels. Another app feature highly preferred by the participants was visual analytics that could present behavioural and health data progress in a graphical format. This feature increases the level of awareness and encourages accountability for self-care behaviours [49, 50], and also provides support for healthy lifestyle decisions [51].

### Recommendations to foster long-term engagement with apps

Ye and his colleagues (2018) reviewed about 1050 apps from the Google play and iTune stores and observed that more than 70% of these apps were designed to support healthy eating mainly with components for ‘carbohydrate count’ and ‘diet tracking’[52]. The present study shows that participants advocated for more comprehensive diet management features in future apps, comprising of nutrient data base of both the common and rare foods, and recipes for tasty foods which are suitable for people with diabetes. Patients’ access to ideas on diverse food varieties via an app, will prevent monotonous dieting and subsequently may foster motivation to maintain a healthy diet and keep track of foods eaten and at the same time boost engagement with the app.

As voiced by the participants, future diabetes apps could include reminders for self-care behaviours which are accompanied by advice on realistic actionable steps to attain those behaviours. App developers could ensure that the proposed actions are simple and instinctively written in plain language, because these are the key factors to attaining health literacy [53]. Additionally, in such suggested steps, users should be able to find what they need, understand what they find and act on it [54].

Participants with type 1 DM recommended the inclusion of community/social forum in apps and this is worth considering by app developers. Type 1 DM is usually diagnosed at a younger age, its management could be quite complex, and requires lifelong daily health commitments. Providing social support for type 1 diabetes patients may improve treatment adherence by encouraging optimism, which can mitigate the stress of living with the illness and accompanying depression [55]. App developers could design apps that offer peer interactions among individuals who share common health experiences [56]. Such social networking features may foster a sense of connection, serve as an essential social support system for patients to be more aware of the importance of diabetes self-management and learn new practical and effective ways for health maintenance. Nevertheless, it is important that patients who use social support features in health apps are reminded not to rely heavily on advice from other users or substitute such information for regular check-ups with their health care providers.

Furthermore, consolidation of data from peripheral devices into app (as proposed by participants), if done appropriately eliminates redundancy and provides data portability. Although in reality, meeting this need will be difficult and may face some challenges particularly when data streaming are from health devices developed by different companies. Such challenges will include which developer will be responsible for the accuracy, reliability, security and compliance with privacy regulation of the consolidated data. This may be the reason why data has remained confined to their respective platforms and integration with other health devices has been very limited [57].

Earlier studies on the use of smartphone apps for health-care have reported ease of use and simplicity as strong determinants of technology acceptance [13, 58] and our study corroborates this finding. Hence, in order to maximize patients’ uptake and long-term engagement with app resources, app developers need to facilitate easier maneuvering through apps menus as a means of reducing the burden of time consumption on users [3].

Customisation of apps in an adaptable way to match the interests, needs or habits of their respective users was another key point raised by the type 1 DM participants in this study. This proposition might have been influenced by participants’ awareness of the highly individualized demand for self-management of type 1 DM [59]. Customizing apps features such as personalized alerts modified to users’ specific needs and preferences may promote the required behavioural change [60] and engagement with the app [31].

### Educational topics in future apps

Problem solving may be a difficult area of diabetes management hence the suggestion by many participants for the inclusion of tips on problem solving in diabetes apps. Problem solving in diabetes management is a learned behavioural process comprising a set of potential solutions to problems, selecting the most appropriate solution, applying the solution and evaluating its effectiveness [61]. Problem solving is the foundation upon which patient attainment of the remaining self-management behaviours (healthy eating, monitoring, reducing risk) are built [62] and there exists a direct correlation between it and improvement in glycosylated hemoglobin (HbA1c) level [63, 64], therefore it is important that patients receive additional support in this area. The support will involve development and incorporation of diabetes specific problem solving measures into apps. Nevertheless, it should be noted that in some situations, apps may not be able to provide effective support to resolve health dilemma in people with diabetes because there may be too many variables in the decision making process needed to address certain challenging areas of self-care, therefore requiring direct interaction between a patient and the clinician or diabetes educator [52]. Moreover, meeting this particular patients’ desire should be handled with caution as diabetes management is uniquely complex, so the use of a single problem solving idea to serve all patients may not be feasible or applicable to some areas of diabetes management.

Personalised or general education is currently available in only a few diabetes apps [2, 17], and participants of the current study, requested for its inclusion in apps. There is ample evidence that patients retain little of the education provided at clinic visits [65]. Since, mobile devices have become ubiquitous in educational settings, they can give expanded opportunities for users to access health information anywhere or double check knowledge [66, 67]. Embedding apps with education and behaviour change techniques can complement instructions provided face to face by health care team members. However, apps that focus on provision of diabetes education should consider the medical accuracy of their content.

### Practical implications

Patients’ recommendations observed in this study demand thoughtful considerations from designers and developers to build mobile apps that best meet the identified patient preferences and propositions. Based on the findings of this study, apps with features on documentation (blood glucose and physical exercise log), reminders (with actionable notifications), advisory (educational information), analytics (view trends in behavioural and health data indicators) and comprehensive nutritional data base are proposed to strengthened the functionality of the apps and foster long-term engagement and better health outcomes for people with diabetes.

Meeting the recommendation for future apps to include a data consolidation system across several peripheral health devices will require careful consideration of existing privacy regulations as this is one of the factors contributing to a lag in data interoperability [57]. Furthermore, the concept of providing a link to social support groups also warrants consideration especially if the app is targeted at people with type 1 DM although, social support can also promote self-care behaviours in those with type 2 DM. Lastly, it is important to ensure ease of use and limited time consumption in the developed app in order to further promote engagement.

It is imperative to evaluate the functionality of developed and operational apps based on experimental pilot studies and large randomized controlled trials (RCTs), which are essential next steps to ascertain patient engagement with the app. This will offer the opportunity to investigate if user engagement can be guaranteed if they (users) are provided with apps that offer the type of features they ‘ask’ for. These experimental studies should aim to integrate app interventions seamlessly into the daily routine of end-users so that app usage is not perceived as an extra chore.

### Strengths and limitations

Firstly, this study was able to quantitatively characterise the social and health demographic profiles of adults with type 1 or type 2 DM in relation to their use of apps and feature preferences. Secondly, through a qualitative approach, it provided information on educational topics which could be included in apps to support diabetes management, in addition to ways to foster adoption and engagement with such apps. To the best of our knowledge, obtaining the information described above in a single study has not been attempted before. Thus, by using a mixed method design which offers the opportunity to comprehensively address the research questions and provide a clear picture of how to meet the research goal; this study fills important knowledge gaps, adds to the body of science and provides direction for future development of functional apps for diabetes self-management. The reliability of the study findings is further strengthened by the fact that participants were asked to indicate their preferences and provide recommendations for a future diabetes app and not on an already developed app. This affords them the convenience to give their candid opinion without the agitation of negatively criticising the work of a developer/investigator.

The findings of this study should be interpreted with the following limitations. Firstly, due to the cross sectional nature of data collection which was done at one point in time, it is possible that people with DM may vary their preferences and recommendations for future diabetes apps over time. Secondly, generalization of the findings to other settings might be limited due to the sample size/groups which mainly comprised of participants from three continents whose responses might have been influenced by cultural or geographic differences. The inclusion of more participants especially from other continents might have led to the emergence of other results. Thirdly, it is worth noting that the quantitative part of this study did not access the differences in preferences of participants based on the type of diabetes they have or the use/non-use of insulin as part of their treatment regimen. Participants’ preferences for app features may be influenced by their treatment therapies or the type of diabetes they have. Fourthly, more themes and topics may have emerged if the interview duration was longer. Given that no additional compensation was offered to interviewee, short interview duration was utilised to foster increased participant numbers as long interview may not be justifiable for participants’ time involvement in the study”. Nevertheless, literature has also shown that telephone interview sessions are usually shorter than face-face interviews. Additionally, even when data saturation was observed at the 12^th^ interview, all the 16 respondents were interviewed to ensure no point was accidentally missed. Lastly, the survey responses were based on self-report and therefore may be subjective to responder’s bias.

### Conclusion

This study will serve as a useful guide for researchers or developers of future apps aimed at diabetes self-management because it highlights the importance of involving end users in the process of developing the content and features that appeal to them. Perceived importance and preferences were higher for app features that could support healthy eating, blood glucose and physical activity monitoring; provide data analytics and diabetes education. Recommendations to foster long-term engagement with diabetes apps have been described via four themes: improved functionalities (exciting and new recipes, actionable goals with convenient reminder), certified and reliable information sources (social networking, research and news update), consolidated and customized features, and ease of use. Additionally, patients are interested in apps embedded with educational information on problem solving techniques, use of insulin pump therapy, signs of diabetes complication and prevention as well as transitioning from paediatric into adult care.

## Supporting information

S1 Checklist Study described according to COREQ criteria. (PDF)

S1 Appendix Survey questionnaire. (PDF)

S2 Appendix Telephone interview guide. (PDF)

## Abbreviations

ANOVA: Analysis of Variance
App: Mobile phone Applications
BG: Blood glucose
DM: Diabetes Mellitus
T1D: Type 1 Diabetes
T2D: Type 2 Diabetes
SD: Standard deviation
UK: United Kingdom
US
