# Supplementary material for: Users’ preferences and design recommendations to promote engagements with mobile apps for diabetes self-management: Multi-national perspectives
Source: PLoS One. 2018 Dec 10;13(12):e0208942. doi: 10.1371/journal.pone.0208942 (PMC6287843; doi:10.1371/journal.pone.0208942)
Supplement: S1 Checklist — (PDF) [file pone.0208942.s001.pdf]

S1 Checklist: Study described according to COREQ Criteria

The below criteria was reproduced from Tong *et al. International Journal for Quality in Health Care*; (2007) 19;6:349-357 according to *PLOS One* submission guidelines.

Domain 1: Research Team and Reflexivity

Personal Characteristics

1. Interviewer/Facilitator – Interview was conducted in Australia by a male pharmacist experienced in qualitative research. The interviewer was not part of the authors but was trained on the aim of the study and the interview guide by the first researcher (MDA). Additionally, a pilot session of the interview was done between the interviewer and MDA.
2. Credentials – The interviewer holds a *MPharmPH*; Authors: MDA: *BSc, MSc, GradCert Diab Edu*; UHM: *MBBS, MSc, MD*; AEOMA: *Bsc, MSc, PhD;* BSMA: *BSc, Msc, GradCert ULT, GradCert Mgt, PhD.*
3. Occupations of research team:
   - Mary D Adu: PhD Candidate, College of Medicine and Dentistry, James Cook University.
   - Usman H Malabu: Consultant Endocrinologist and Professor of Medicine, Townsville Hospital and Health Services / College of Medicine and Dentistry, James Cook University.
   - Aduli EO Malau-Aduli: Associate Professor, College of Public Health, Medical and Veterinary Sciences, James Cook University.
   - Bunmi S Malau-Aduli: Associate Professor, College of Medicine and Dentistry, James Cook University.

4. Gender – By author: MDA: Female, UHM: Male, AEOMA: Male, BSMA: Female
5. Experience and Training: All authors were experienced researchers and have taken part in several primary researches and published peer-reviewed literature in mobile applications for diabetes management.

Relationship with Participants

6. Relationship Established – Interviewer had no prior relationship with the participants. All participants had the opportunity to ask questions and express concerns during the consent-taking process.
7. Participant Knowledge of the Interviewer – The background and aim of the study were explained to the participants during the introduction to the interview and the consenting process (see S2 Appendix)
8. Interviewer Characteristics – The interviewer was only involved in the qualitative study. He specifically assisted in conducting the interviews and did not participate in other study procedures.

Domain 2: Study Design

Theoretical Framework

9. Methodological Orientation and Theory – As described in the manuscript, qualitative description methodology was utilized.

Participant Selection

10. Sampling – A convenient sampling was utilized. Only those who provided their phone numbers through the interview to indicate interest in participation were contacted.
11. Method of Approach – Participants were a subset of those who participated in an online survey and indicated interest to participate in the interview by providing their best contact numbers. They were initially sent a text message by MDA to confirm interview schedules.
12. Sample Size – 16 participants.
13. Non-Participation – 31 participants initially indicated interest to participate, 15 subsequently declined or did not pick their calls after several attempts.

Setting

14. Setting of Data Collection – The interviewer was located in a secure, private room at the James Cook University, Australia. Prior to the commencement of each interview session, participants were asked if they were in a location that was convenient for the interview.
15. Presence of Non-Participants – Author MDA was present in the first three interviews to listen to the interaction between the participants and the interviewer and to ensure appropriate data acquisition. MDA had no conversation with those participants.
16. Description of Sample – Please see the results session, pages 13.

Data Collection

17. Interview Guide – Please see S2 Appendix.
18. Repeat Interviews – No repeat interviews were required.
19. Audio/Visual Recording – Data was collected via audio recording. No visual recording was done
20. Field Notes – None.
21. Duration – Each interview lasted approximately 7 and 20 minutes
22. Data Saturation – Data saturation was achieved at the end of the 12th interview, that is, the information was deemed sufficient as there were no new response patterns identified by the interviewer at this point. However, interview sessions with all consenting respondents (16) were completed in order to allow rich documentation and to ensure no point was accidentally missed.
23. Transcripts Returned – Given participants' remoteness from the research site, transcripts were unable to be returned to participants for comments.

Domain 3: Analysis and Findings

Data Analysis

24. Number of Data Coders – Two. Please check full details on page 11.
25. Description of the Coding Tree – Please see the qualitative data analysis session, page 11.
26. Derivation of Themes – Themes were inductively derived from the data rather than in advance.
27. Software – Data was managed using Microsoft Word and QSR Nvivo 11 qualitative analysis software.
28. Participant Checking – As noted above, given participants' remoteness from research site, the findings were unable to be presented to the participants for comments.

Reporting

29. Quotations Presented – Quotations were presented in the manuscript to illustrate each theme.
30. Data and Findings Consistent – There was consistency between the data presented and the findings.
31. Clarity of Major Themes – Major themes were presented in the body of the manuscript.
32. Clarity of Minor Themes – Minor themes were presented in the body of the manuscript.
