# Supplementary material for: Users’ preferences and design recommendations to promote engagements with mobile apps for diabetes self-management: Multi-national perspectives
Source: PLoS One. 2018 Dec 10;13(12):e0208942. doi: 10.1371/journal.pone.0208942 (PMC6287843; doi:10.1371/journal.pone.0208942)
Supplement: S1 Appendix — (PDF) [file pone.0208942.s002.pdf]

S1 Appendix: Survey Questionnaire

Introductions

Welcome to this survey and thank you in advance for your interest to participate.

This survey aims to examine the use and preferences of features in mobile apps to manage diabetes. The result of this study will help in gaining better understanding of the important features and components to consider before developing future apps to manage diabetes. Your responses, including your demographic information will remain anonymous. At the end of the survey, you will be asked if you are interested in participating in an additional telephone interview. If you choose to provide your phone number, your responses to the phone interview may not be anonymous to the researcher. However, you will not be identified in any way in the research publications and results from this study.

Eligibility

Only people who have type 1 or type 2 diabetes and are 18 years of age or above are eligible to participate in this survey. If you do not meet any of these criteria please exit the survey.

Instructions on completing the survey

Please read and consider the following questions carefully. Your candid responses are essential to ensure the reliability of this study.

Section A: Demographic

Below are questions about your background. Please pick one response for the multiple choice question

1. **What is your gender?**

   ☐ Male ☐ Female ☐ Others (Pls. specify) ……………..

2. **How old are you?** _____________(years)

3. **What is the highest level of education you have completed?**

   ☐ High school or equivalent ☐ Technical or vocational education

   ☐ College ☐ Bachelor degree ☐ Post graduate degree

   ☐ No formal education ☐ Others

4. **What is your employment status?**

   ☐ Employed ☐ Unemployed ☐ Retired

5. **What continent do you presently live in or reside?**

   ☐ America ☐ Asia ☐ Australia ☐ Europe

   ☐ Africa ☐ Antarctica

6. **What type of diabetes do you have?**

   ☐ Type 1 ☐ Type 2 ☐ Gestational

   ☐ I don't have diabetes ☐ Others (Please specify) ………………………….

7. **How long have you been diagnosed with diabetes (years)?**

   ☐ < 1 ☐ 1- 5 ☐ 6 - 10 ☐ 11 -15 ☐ ≥ 15 years ☐ Never

Section B

The next set of questions are related to your use of mobile phone health applications for your diabetes management. Mobile phone application (app) is any program on a smart phone designed to support any aspect of your diabetes management to foster improved health outcomes. Some apps aid in tracking or managing blood sugar, diet, blood pressure; there are also some for reminding you about taking your medications.

1. Do you currently have apps on your mobile phones that provides support related to your diabetes management?

   Yes ☐ **(please go to question 2)**

   No ☐ **(please go to question 5)**

2. If yes, what are the features in the apps available on your smart phone which you use to manage your diabetes (tick all that applies to you).

   ☐ Blood pressure tracker

   ☐ Blood glucose tracker

   ☐ Reminder (e. g to take medication or attend a health appointment)

   ☐ Fitness / exercise monitor

   ☐ Body weight tracker

   ☐ Food calorie counter

   ☐ Feature to transfer electronic health information to your doctor

   ☐ Others (Please Specify……………………………………………)

3. **Which of these features in apps do you use regularly? Only tick the ones you use at least 4 times in a week**

☐ Blood pressure tracker

☐ Blood glucose tracker

☐ Reminder (e. g to take medication or attend a health appointment)

☐ Fitness/ exercise monitor

☐ Body weight tracker

☐ Food calorie counter

☐ Feature to transfer electronic health information to your doctor

☐ Others (Please Specify……………………………………………)

4. **Are there specific reasons why you don't use other features regularly**

a. ……………………………………………………………………………………..

b. ……………………………………………………………………………………..

c. ……………………………………………………………………………………..

5. **If No to question 1 of this section, which of the following is a reason for not using apps to support your diabetes management (tick all that applies to you).**

☐ I am not interested

☐ I am not aware

☐ Lack of smart phone

☐ It is expensive

☐ Limited access to the internet

☐ Others (please specify…………………………………………..)

6. Please rate each of these app features by indicating the extent to which you think they are useful to support your diabetes management. Pick only one number for each of the app feature. Score 10 being the most useful and 1 being the least useful.

**Health Applications**	**Perceived order of usefulness**									
	**Most useful**									**Least useful**
	**10**	**9**	**8**	**7**	**6**	**5**	**4**	**3**	**2**	**1**
Blood pressure tracker	☐	☐	☐	☐	☐	☐	☐	☐	☐	☐
Blood glucose tracker	☐	☐	☐	☐	☐	☐	☐	☐	☐	☐
Food nutrient composition	☐	☐	☐	☐	☐	☐	☐	☐	☐	☐
Fitness / exercise monitor	☐	☐	☐	☐	☐	☐	☐	☐	☐	☐
Body weight monitor	☐	☐	☐	☐	☐	☐	☐	☐	☐	☐
Data transfer to health care team (e. g doctor)	☐	☐	☐	☐	☐	☐	☐	☐	☐	☐
Task reminder app (e.g to take medication, appointment with doctor)	☐	☐	☐	☐	☐	☐	☐	☐	☐	☐

7. **If offered the option of using a new mobile diabetes application (app) to support your diabetes management? Please choose the features you would like the app to have. (Tick all that applies to you).**

☐ Provide access to nutritional information on nutrient contents of foods

☐ Log blood glucose levels

☐ Physical exercise tracker

☐ Task reminders (e.g reminder to take my medication)

☐ General information on diabetes; its management and complication prevention

☐ Graphical display of logged data

☐ Logs and display of body weight maintenance

☐ Feature that allows me to network with other people having diabetes

8. Are there other suggestions or improvements that is not listed which you think are very important in apps that support the management of diabetes

…………………………………………………………………………………………

…………………………………………………………………………………………

…………………………………………………………………………………………

…………………………………………………………………………………………

Would you be interested to be contacted for an individual interview session? The session is to hear your experience on managing diabetes and your opinion on how mobile phone apps could be improved to manage diabetes and some of the important components to include in future apps for diabetes management. If yes, please indicate and provide your phone number and best time to contact you.

☐ Yes

Phone number…………….Best time to contact…………………………

☐ No
