# Supplementary material for: Users’ preferences and design recommendations to promote engagements with mobile apps for diabetes self-management: Multi-national perspectives
Source: PLoS One. 2018 Dec 10;13(12):e0208942. doi: 10.1371/journal.pone.0208942 (PMC6287843; doi:10.1371/journal.pone.0208942)
Supplement: S2 Appendix — (PDF) [file pone.0208942.s003.pdf]

S2 Appendix: Interview guide

Introduction of study and interviewer:

Hello, my name is ……………..I’m calling in regards to an online questionnaire that you participated in a couple of months ago relating to diabetes management and the use of mobile phone application (app). You left a phone number to indicate your interest in participating in a follow up interview to the survey and a text was sent to you few days ago. The interview will take about 10 minutes, is it ok to proceed with the interview now or you would like me to call back at a later time?

Thank you for your willingness to participate and be interviewed. Please note that I will be recording this interview, and it will be transcribed and analysed. You are free to choose what to disclose for any of your answers to the questions or refuse to answer a particular question. The aim of the interview is to gather information on how apps could be improved to manage diabetes. An app is any program downloadable to a smart phone which is used to support any aspect of your diabetes management to foster improved health outcomes. It could be an app for lifestyle monitoring such as those for tracking diet and exercise or for monitoring your clinical outcomes such as blood glucose or blood pressure.

We have observed that often times, when a lot of people with diabetes use apps to manage any aspect of their health, their interest in using the app decreases over time. I would like to explore your opinions on what to include in apps to prevent such decrease in usage over time.

1. Do you currently use an app to support your diabetes care?

   a. If yes, what are the ones you use/what aspect of your diabetes care do they support?
   b. If no, have you in time past used any?
   c. If yes to question b above, what are your reasons for stopping? (if no, reasons for non-usage)?

2. What do you think could be the reason why people with diabetes use an app for a period of time with a subsequent decrease / stoppage in its use over time?

3. What do you think would be a motivator to encourage you to continually use an app for your diabetes management?

4. Is there any feature or function you would consider very important for inclusion in a good app which could stimulate your interest to use it regularly?

5. What are possible improvements in app features that might motivate you to use it regularly?

6. Are there other aspects/components/features you would like to see in future apps which could encourage you to use it more frequently and regularly? What are your expectations of future apps to manage diabetes?

For all questions involving app features, there were specific probes on features for blood glucose monitoring, activity tracking, reminder, features on nutrition to foster healthy eating, etc.)

Educational topics desired in Diabetes Apps

1. When it comes to diabetes and its management, what sort of education or information would you desire to be included in future apps to support diabetes management? (Specifically probe on healthy eating, preventing diabetes complications, risk reduction, engaging in physical activities, adherence to medication intake, responding to challenges of diabetes clinical outcomes and self-management).
